# Feasibility of Preoperative Video Head Impulse Test to Predict the Nerve of Origin in Patients with Vestibular Schwannomas

**DOI:** 10.3390/jcm10122677

**Published:** 2021-06-17

**Authors:** Gi-Sung Nam, Seong-Hoon Bae, Hye-Jeen Kim, Ji-Woong Cho, In-Seok Moon

**Affiliations:** 1 Department of Otorhinolaryngology, Chosun University College of Medicine, Gwangju 61453, Korea; entnamgi@gmail.com (G.-S.N.); digginonyou77@hanmail.net (H.-J.K.); woonghi1@naver.com (J.-W.C.); 2 Department of Otorhinolaryngology, Yonsei University College of Medicine, Seoul 03722, Korea; bshsap@naver.com

**Keywords:** vestibular schwannoma, vestibular function test, video head impulse test, superior vestibular nerve, inferior vestibular nerve

## Abstract

Vestibular schwannoma (VS) originates from Schwann cells in the superior or inferior vestibular nerve. Identifying the precise origin will help in determining the optimal surgical approach. We retrospectively analyzed the preoperative vestibular function test according to VS origin to determine whether the test is a valuable indicator of tumor origin. Forty-seven patients with VS (male:female = 18:29, mean age: 54.06 ± 13.50 years) underwent the cochleovestibular function test (pure-tone audiometry, caloric test, video head impulse test (vHIT), cervical and ocular vestibular-evoked myogenic potential, and posturography). All patients then underwent surgical removal of VS, and the schwannoma origin was confirmed. The tumor originated from the superior vestibular nerve (SVN group) in 21 patients, the inferior vestibular nerve (IVN group) in 26 patients, and an undetermined site in eight patients. The only value that differed significantly among the groups was the gain of the vestibular-ocular reflex (VOR) in the ipsilesional posterior canal (iPC) during the vHIT. Our results indicate that VOR gain in the iPC may be used to predict the nerve origin in patients with VS. Other cochleovestibular function tests have limited value to discriminate nerve origins, especially in cases of medium to large VS.

## 1. Introduction

Vestibular schwannoma (VS), also known as acoustic neuroma, is a benign, slow-growing tumor at the glial-Schwann cell junction of the vestibular nerve [1,2]. The majority of VS originates from one of the two vestibular divisions of the eighth cranial nerve: the superior vestibular nerve (SVN) or the inferior vestibular nerve (IVN); less frequently, it originates from the cochlear or facial nerves [3]. The goal of VS surgery is to achieve complete removal of the tumor with minimal postoperative morbidity and mortality [4]. This can be achieved by accurately predicting the origin of VS, as hearing preservation is often better for VS arising from the SVN [4,5,6,7,8]. Thus, preoperative identification of the VS origin is of great interest for surgeons. Knowledge of the nerve origin will help determine the optimal surgical approach [4,9].

Given the dependency of the vestibular nerve function, the SVN transmits the signal of the anterior canal, horizontal canal, and utricle, and the IVN transmits the signal of the saccule and posterior canals. Each integrity can be measured indirectly through the caloric test and ocular vestibular-evoked myogenic potential (oVEMP) for the SVN and cervical vestibular-evoked myogenic potential (cVEMP) for the IVN [10]. Therefore, several attempts have been made to preoperatively predict the origin using various vestibular function tests, including the caloric test, cVEMP, and oVEMP [6,11,12,13]; however, these attempts have been unsuccessful or limited, particularly in cases of large tumors. The video head impulse test (vHIT), a relatively new vestibular-ocular response (VOR) test method, can quantitatively measure the VOR gain, as well as identify saccades on the horizontal canals (HCs), anterior canals (ACs), and posterior canals (PCs) [14]. However, there is limited information regarding the function of the three semicircular canals using vHIT, which may determine the origin of VS [15,16,17].

In most cases, the origin of VS can be determined intraoperatively by an experienced surgeon. Thus, the primary purpose of this study was to retrospectively analyze preoperative vestibular function tests according to VS origin, which was confirmed intraoperatively, to determine whether the various vestibular function tests can be used to distinguish tumor origin.

## 2. Materials and Methods

### 2.1. Patients

Medical records of 55 patients who underwent VS surgery in a single tertiary hospital between March 2019 and August 2020 were retrospectively reviewed. Among the 55 patients, the nerve origin of the tumor in eight patients could not clearly be identified during the surgery; therefore, they were not included in further analysis. Consequently, 47 patients were enrolled in the present study. This study was approved by the institutional review board of Severance Hospital in Seoul, Korea (project number 4-2021-0089). As this was a retrospective study, the requirement for informed consent was waived.

### 2.2. Tumor Evaluation

High-resolution axial T2-weighted FIESTA sequences were used to measure tumor size, defined as the maximal diameter of the extrameatal portion in the axial magnetic resonance imaging (MRI) results. Tumor grade was defined by Koos grade [18] and Hannover classification [19] based on the MRI findings (Table 1). The tumor subclassification (cystic or solid type) was completed by a specialized radiologist; a cystic tumor was defined when the cystic portion exceeded 50% of the tumor volume. The origin of the tumor was determined in the operation room by a single experienced surgeon (more than 300 cases of skull-base surgery, I.S. Moon), and histopathology was used to confirm whether the tumor was a schwannoma. The surgeon was uninformed about the results of vestibular function tests before and during the surgery.

### 2.3. Pure-Tone and Speech Audiometry

The pure-tone and speech audiograms were completed for all enrolled patients by an experienced audiologist before surgery. The average pure-tone thresholds of 500, 1000, 2000, and 4000 Hz in the affected ear were defined as PTA_4_. The speech discrimination score was defined as the percentage of correctly repeated 50 monosyllabic Korean words in the sound intensity of the most comfortable hearing level. For both tests, the audiologist used noise masking as needed in the patient’s unaffected ear.

### 2.4. Caloric Test

Binaural bithermal caloric irrigations (SLVNG, SLMED, Seoul, Korea) were performed with the patient positioned in a chair reclined 30° to vertically orient the semicircular canals. The external auditory canal was irrigated with 20 mL of cold water at 30 °C, followed by 20 mL of hot water at 44 °C for 10 s after confirming the integrity of the tympanic membrane. The induced nystagmus was recorded using electronystagmography in a dark room with the patient’s eyes open, and a maximum slow-phase velocity of nystagmus was measured following each irrigation. Canal weakness was defined as a > 25% asymmetry based on Jongkees’ formula [20].

### 2.5. Cervical and Ocular Vestibular-Evoked Myogenic Potentials (cVEMP and oVEMP)

We measured cVEMPs and oVEMPs using the Audera system (Audera, GSI, Eden Prairie, MN, USA) with short-tone bursts from earphones at a frequency of 500 Hz with an amplitude of 100 dB. The cVEMPs were registered using surface electrodes placed on the upper half of the ipsilateral sternocleidomastoid muscles, a reference electrode on the mastoid, and a ground electrode on the forehead. The first positive–negative peak (p13–n23) of the averaged electromyogram results was analyzed and recorded as the cVEMP amplitude. For oVEMP, the electrode was placed directly beneath the patient’s eye contralateral to the forehead ground electrode, and acoustic stimuli were applied to the contralateral ear. The n10–p15 amplitudes of the averaged electromyogram results were evaluated and recorded as the oVEMP amplitudes. Asymmetry ratios less than −0.36 or no response were considered abnormal responses, calculated as follows: (ipsilateral amplitude—contralateral amplitude)/(ipsilateral amplitude + contralateral amplitude) at 100 dB nHL [21].

### 2.6. Video Head Impulse Test (vHIT)

The function of the six semicircular canals was evaluated using a three-dimensional video head impulse test (vHIT, ICS Impulse, Otometrics, Taastrup, Denmark). Patients were positioned at a distance of 1 m from a target located at eye level. To ensure reliability of the examination process, goggles were fastened to the patient’s head with an elastic band to minimize slippage. The examination was conducted by an experienced technician blinded to the results of the clinical and MRI findings. The technician manually performed rotation more than 20 times (head rotation: 15–20°, duration: 150–200 ms, peak velocity: >150°) on both sides of each plane. The VOR gain of the three semicircular canals was calculated using the ratio of the area under the curve (AUC) for the eye velocity area divided by the head velocity area, as automatically determined by the device. All vHIT tests were conducted by a senior ENT technician blinded to the results regarding tumor origin. The overt or covert saccades used in the analysis were defined as the occurrence of more than 20% of trials with similar amplitude and latency during all vHIT trials.

### 2.7. Posturography

Posturography was performed on a computerized dynamic posturography platform (EquiTest; Neurocom, Clackamas, OR, USA). A composite (COMP) score was calculated by independently averaging the equilibrium score for conditions 1 and 2, adding them to the equilibrium score from each trial of conditions 3–6, and finally dividing this sum by the total number of trials. The ratio of C5 to C1 represented the vestibular contribution (VEST). The detailed analysis methods were previously described [22].

### 2.8. Statistical Analysis

All statistical analyses were performed using the Statistical Package for the Social Sciences for Windows version 20.0 (IBM Corp., Armonk, NY, USA). A *p*-value < 0.05 was considered statistically significant. Quantitative parameters were described using means ± standard deviations (SDs). The independent *t*-test, Mann–Whitney U test, Pearson or Spearman correlation analysis, and Fisher’s exact test were used to compare parameters in this study. Each evaluation method was selected based on the normality of the sample. Receiver operating characteristic (ROC) curve analysis was performed to identify which parameters were useful in distinguishing SVN and IVN groups.

## 3. Results

### 3.1. Demographic Data of Patients and Surgical Results

Patient demographics and tumor characteristics are outlined in Table 1. Among the 55 enrolled patients who underwent surgical removal of VS, the nerve of the tumor origin in eight of them could not be clearly identified during the surgery; therefore, they were not included in further analysis. Forty-seven patients were finally included, with a mean age of 54.06 ± 13.50 years and a male to female ratio of 18:29. Tumor origin was identified by a single experienced neuro-otologic surgeon (I.S. Moon). Tumors arose from the SVN in 21 patients and the IVN in 26 patients. The mean extrameatal tumor size was 19.62 ± 8.88 mm, with 19.33 ± 10.00 mm for SVN origin and 19.85 ± 8.05 mm for IVN origin. There were no significant differences between the SVN and IVN groups regarding tumor characteristics, including tumor size, Koos grade, Hannover classification, cystic component, or surgical approach (Table 1).

### 3.2. Cochleovestibular Function According to Tumor Origin

**Figure 1:**
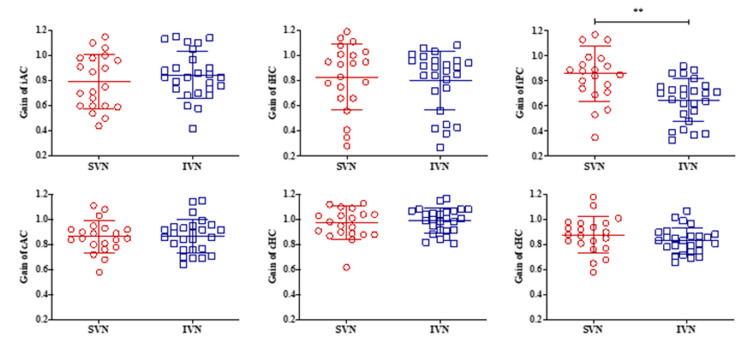
The VOR gain of the ipsilesional (i) and contralesional (c) sides of three semicircular canals in patients with vestibular schwannomas. Compared with the SVN groups, the VOR gain for the IVN groups was reduced significantly only in the iPC; ** *p*-value < 0.01; SVN, superior vestibular nerve; IVN, Inferior vestibular nerve; AC, anterior canal; HC, horizontal canal; PC, posterior canal; i = ipsilesional; c = contralesional.

The PTA_4_ was 52 dB HL, with 40% speech discrimination in the SVN group and 60 dB HL with 45% speech discrimination in the IVN group. The mean caloric weaknesses were 42% and 44% in the SVN and IVN groups, respectively. The mean VOR gain on the ipsilesional side in the SVN group was 0.79 (SD = 0.22) in the AC, 0.83 (SD = 0.26) in the HC, and 0.86 (SD = 0.22) in the PC. For the IVN group, the VOR gain was 0.85 (SD = 0.19) in the AC, 0.80 (SD = 0.23) in the HC, and 0.65 (SD = 0.17) in the PC. We observed statistical differences in VOR gain only in the ipsilesional PC (iPC) (*p* = 0.001) (Table 1 and Figure 1). During cVEMPs testing, 16 patients (76.2%) in the SVN group and 21 (80.8%) in the IVN group had abnormal responses. During oVMEPs testing, this was seen for 18 patients (85.7%) in the SVN group and 23 (88.5%) in the IVN group. The abnormal response rates for cVEMPs and oVEMPs did not differ significantly between the two groups (Table 2). In addition, no statistical differences were observed in the composite scores or vestibular scores of posturography (Table 2). Vestibular function in relation to tumor origin is also shown in Table 2.

### 3.3. vHIT to Identify the Nerve of Origin of VS

**Figure 2:**
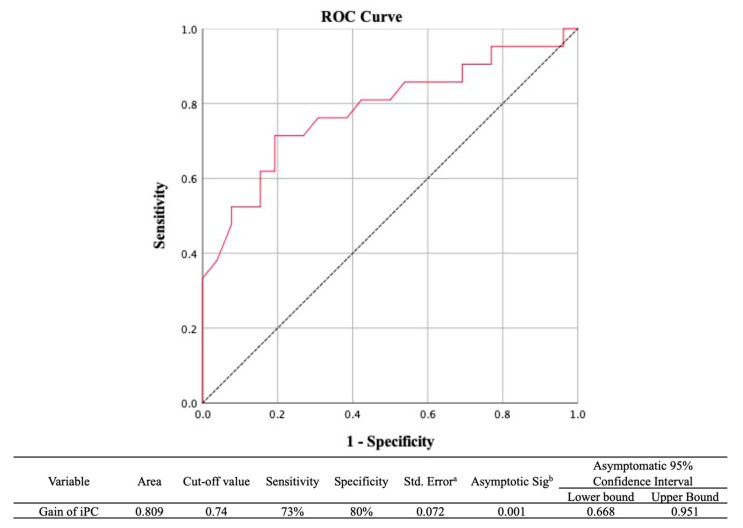
ROC curves for the IVN groups in the iPC gain to predict differentiation of IVN from SVN. The optimal cutoff value for gain in the iPC provides the sensitivity (73%) and specificity (80%). The black dashed line indicates a hypothetically useless diagnostic test. ROC, receiver operating characteristic; SVN, superior vestibular nerve; IVN, inferior vestibular nerve; PC, posterior canal; i = ipsilesional. a. Under the nonparametric assumption. b. Null hypothesis: true area = 0.5.

We plotted the ROC curve to determine whether VOR gain in the iPC had diagnostic value in discriminating tumor origin. The AUC of the iPC was 0.801, indicating a good parameter [23]. Tumors originating from the IVN could be diagnosed with a sensitivity of 73% and a specificity of 80% when the iPC gain was below 0.74 (Figure 2). We also observed that the asymmetry ratio of the PC calculated by Jonkees’ formula could not provide additional diagnostic value in discriminating the two origins (AUC, 0.729).

## 4. Discussion

This study was conducted to determine the differences in vestibular function testing when VS originates from the IVN and SVN by quantitatively evaluating the results of a relatively large number of VS patients for whom the origin was confirmed during surgery. The advantage of a test battery that combines the caloric test, vHIT, and c or oVEMP lies in the ability to identify patterns of nerve and end-organ involvement; vHIT can be used in conjunction with air conduction c and oVEMP to identify four categories that characterize patients with vestibular neuritis: SVN, IVN, both nerve divisions, and canal afferents only [10]. Horizontal vHIT gain, the caloric test results, and oVEMP responses reflect the superior afferent function, and posterior canal vHIT and cVEMP responses reflect the inferior afferent function [10].

Based on these findings, we believe that the nerve origin of VS can be predicted using vestibular function tests, including the caloric, vHIT, and VEMP tests. Since the origin of the tumor is an important prognostic factor, several attempts have been made to predict it with various vestibular function tests, hearing status, and by using MRI [6,12,13,15,16,24]. Due to the close relationship between the tumor and cochlear nerve under the transverse crest within the internal auditory canal, removal of VS originating from the IVN is considered to be responsible for the low rate of hearing preservation, but it is also associated with an increased chance of intraoperative trauma to the cochlear nerve [5,9]. This association is supported by several studies demonstrating that 61% to 75% of patients whose tumors were found intraoperatively to arise from the SVN had preserved hearing, while hearing was preserved in only 16% to 28% of patients whose tumors were found to arise from the IVN and for whom hearing preservation was attempted [5,6]. This information is useful in that it further enables surgeons to determine the best surgical approach and accurately counsel patients preoperatively based on their test results. However, most studies to preoperatively predict the origin using functional vestibular tests have been unsuccessful; even when they were successful, they were limited to relatively small tumors. As such, intraoperative identification remains the only reliable method [15,16].

While deteriorated vestibular function could potentially be associated with a tumor arising from the SVN or a large tumor arising from the IVN, a normal or localized abnormal vestibular response is considered to indicate a small tumor. Therefore, the need to adequately discern the origin of small tumors becomes somewhat reasonable according to the vestibular functions [12,16,24]. However, when the tumor size exceeds medium and most vestibular functions are deteriorated [15], the simple pathologic classification has limitations in determining the nerve origin; in some cases, it is not even possible to make a prediction. Therefore, we quantitatively evaluated vestibular functions, especially in vHIT, at three semicircular canals in patients with schwannomas for whom the origin was identified during surgery. Our findings demonstrated significant differences in gain in the iPC, depending on the tumor origin (0.86 ± 0.22 in SVN and 0.65 ± 0.17 in IVN, *p* = 0.001). In addition, the ROC curve showed that the difference of PC gain can distinguish the origin of the tumor with a sensitivity of 73% and a specificity of 80% (Figure 2). However, other vestibular function tests, including the caloric weakness, abnormal rate of the VEMP test, and postural score, showed no differences between the two groups. Similar to our results, Inoue et al. reported that the caloric test did not provide information regarding nerve origin [24]. The specific value of VEMP in predicting outcomes varies among reports. He et al. reported that SVN tumors showed an abnormal cVEMP response in about 50% of cases, whereas, in IVN tumors, no or decreased cVEMP response was observed in all cases [6]. Chen et al. also reported that, in patients with normal caloric responses and no VEMP, the VS originated from the IVN [25]. The differences between our study and others on VEMP indicate that, in VS cases larger than medium size, nerve origin cannot be precisely predicted using conventional vestibular function tests. Other authors have also shown that caloric tests and VEMP responses did not significantly differ between patients with SVN and IVN schwannomas [11,13]. Tsutsumi et al. [12] reported that VEMP was not useful for preoperatively predicting the origin of schwannomas because of its tolerance for minor dysfunction of the inferior vestibular afferent nerve.

Regarding vHIT, several attempts have been made to evaluate the vestibular function related to nerve origin [15,16]. Taylor et al. [26] found little evidence of selective inferior or superior nerve subtypes in 50 patients with VS, but a surgical correlation was not sought. Recently, Rahne et al. [16] attempted to identify nerve origin by providing an independent score for the presence or absence of vHIT and VEMP abnormalities with five VS patients; they were able to correctly predict the origin in four cases. However, the tumors that could be identified by the scoring system were small (Koos grade I, II). Thus, tumor size seems to be a limiting factor for prediction using these scoring systems. Another study demonstrated the usefulness of vHIT to correctly identify the nerve of origin in 89.7% of cases [15]. In this study, the results of vHIT were evaluated using normative values and classified into normal pattern, isolated SVN, isolated IVN, predominant SVN, and predominant IVN. However, in our study, posterior vHIT gain was identified as the only reliable factor that could distinguish the two origins. In most cases, an experienced surgeon can identify the nerve of origin intraoperatively. Therefore, the risk of intraoperative misjudgment was considered very low in our study.

Reliable preoperative identification of the nerve of origin in patients with VS would be useful; it would provide information regarding the chance of hearing preservation when surgery is recommended. Therefore, in a situation where an inferior VS is suspected from vHIT analysis, the retrosigmoid approach, which allows direct visualization of both vestibular nerves on the IAC, may have an advantage for tumor removal, and better hearing preservation can be expected.

Our results indicate that VOR gain in the iPC has a localizing value to predict the nerve origin in patients with VS. Other cochleovestibular function tests have limited value to discriminate nerve origins, especially for cases of large VS.

## Figures and Tables

**Table 1:** Patient demographics and tumor characteristics.

Characteristics		Tumor Origin		
	All Patients (*n* = 47)	SVN (*n* = 21)	IVN (*n* = 26)	*p*-Value
Age	54.06 ± 13.50	53.62 ± 13.40	54.42 ± 13.83	0.84
M:F	18:29	8:13	10:16	1
R:L	19:28	10:11	9:17	0.39
Tumor size	19.62 ± 8.88	19.33 ± 10.00	19.85 ± 8.05	0.85
Koos grade				
I	5 (10.6%)	4 (19.0%)	1 (3.8%)	0.16
II	22 (46.8%)	9 (42.9%)	13 (50%)	0.78
III	17 (36.2%)	7 (33.3%)	10 (38.5%)	0.77
IV	3 (6.4%)	1 (4.8%)	2 (7.7%)	1
Hannover classification				
T1	7 (14.9%)	5 (23.8%)	2 (7.7%)	0.27
T2	8 (17.0%)	1 (4.8%)	7 (26.9%)	0.06
T3a	5 (10.6%)	2 (9.5%)	3 (11.5%)	1
T3b	10 (21.3%)	5 (23.8%)	5 (19.2%)	0.73
T4a	10 (21.3%)	5 (23.8%)	5 (19.2%)	0.73
T4b	7 (14.9%)	3 (14.3%)	4 (15.4%)	1
Cystic component				
Cystic	12 (25.5%)	3 (14.3%)	9 (34.6%)	0.18
Solid	35 (74.5%)	18 (85.7%)	17 (65.4%)	0.18
Surgical approach				
TLA	34 (72.3%)	17 (81.0%)	17 (65.4%)	0.33
MFA	1 (2.1%)	1 (4.8%)	0 (0.0%)	0.11
RSA	8 (17.0%)	2 (9.5%)	6 (23.1%)	0.27
EETTA	4 (8.5%)	1 (4.8%)	3 (11.5%)	0.62

SVN, superior vestibular nerve; IVN, inferior vestibular nerve; M, male; F, female; TLA, translabyrinthine approach; MFA, middle fossa approach, RSA; retrosigmoid approach; EETTA, exclusive endoscopic transcanal transpromontorial approach.

**Table 2:** Cochleovestibular function according to tumor origin.

Parameters		Tumor Origin		
		SVN (*n* = 21)	IVN (*n* = 26)	*p*-Value
Pure-tone audiometry (PTA_4_)		51.90 ± 29.00	59.87 ± 25.38	0.32
Speech discrimination (%)		40.38 ± 35.42	44.67 ± 36.65	0.6
Canal weakness (%)		41.83 ± 27.79	43.84 ± 23.98	0.8
vHIT				
iAC	Gain	0.79 ± 0.22	0.85 ± 0.19	0.36
	Overt saccades	3	2	0.64
	Covert saccades	5	4	0.49
	Any saccades	7	6	0.52
iHC	Gain	0.83 ± 0.26	0.80 ± 0.23	0.72
	Overt saccades	16	17	0.53
	Covert saccades	7	10	0.77
	Any saccades	16	18	0.75
iPC	Gain	0.86 ± 0.22	0.65 ± 0.17	0.001
	Overt saccades	8	17	0.08
	Covert saccades	4	10	0.2
	Any saccades	12	19	0.36
cAC	Gain	0.86 ± 0.13	0.87 ± 0.13	0.89
	Overt saccades	3	0	0.08
	Covert saccades	2	4	0.68
	Any saccades	4	4	1
cHC	Gain	0.98 ± 0.13	0.99 ± 0.10	0.64
	Overt saccades	8	6	0.34
	Covert saccades	1	6	0.11
	Any saccades	9	9	0.76
cPC	Gain	0.88 ± 0.14	0.83 ± 0.10	0.23
	Overt saccades	6	7	1
	Covert saccades	2	5	0.44
	Any saccades	7	9	1
cVEMP (% abnormal responses)		16 (76.2%)	21 (80.8%)	1
oVEMP (% abnormal responses)		18 (85.7%)	23 (88.5%)	1
Posturography				
Composite score		68.25 ± 9.43	60.77 ± 11.99	0.228
Vestibular score		56.00 ± 15.65	39.69 ± 22.69	0.468

SVN, superior vestibular nerve; IVN, inferior vestibular nerve; AC, anterior canal; HC, horizontal canal; PC, posterior canal; i = ipsilesional; c = contralesional; cVEMP, cervical vestibular-evoked myogenic potentials; oVEMP, ocular vestibular-evoked myogenic potentials.

## Data Availability

The original contributions presented in the study are included in the article, further inquiries can be directed to the corresponding author.
